# Integrated lipidomics and proteomics reveal cardiolipin alterations, upregulation of HADHA and long chain fatty acids in pancreatic cancer stem cells

**DOI:** 10.1038/s41598-021-92752-5

**Published:** 2021-06-24

**Authors:** Claudia Di Carlo, Bebiana C. Sousa, Marcello Manfredi, Jessica Brandi, Elisa Dalla Pozza, Emilio Marengo, Marta Palmieri, Ilaria Dando, Michael J. O. Wakelam, Andrea F. Lopez-Clavijo, Daniela Cecconi

**Affiliations:** 1 Mass Spectrometry and Proteomics Lab, Department of Biotechnology, University of Verona, Strada le Grazie 15, 37134 Verona, Italy; 2 Lipidomics Facility, Babraham Research Campus, Babraham Institute, Cambridge, CB22 3AT UK; 3 Department of Sciences and Technological Innovation, University of Eastern Piedmont, 15121 Alessandria, Italy; 4 Department of Neurosciences, Biomedicine and Movement Sciences, University of Verona, 37134 Verona, Italy

**Keywords:** Cancer metabolism, Cancer stem cells, Lipidomics, Proteomics

## Abstract

Pancreatic cancer stem cells (PCSCs) play a key role in the aggressiveness of pancreatic ductal adenocarcinomas (PDAC); however, little is known about their signaling and metabolic pathways. Here we show that PCSCs have specific and common proteome and lipidome modulations. PCSCs displayed downregulation of lactate dehydrogenase A chain, and upregulation of trifunctional enzyme subunit alpha. The upregulated proteins of PCSCs are mainly involved in fatty acid (FA) elongation and biosynthesis of unsaturated FAs. Accordingly, lipidomics reveals an increase in long and very long-chain unsaturated FAs, which are products of fatty acid elongase-5 predicted as a key gene. Moreover, lipidomics showed the induction in PCSCs of molecular species of cardiolipin with mixed incorporation of 16:0, 18:1, and 18:2 acyl chains. Our data indicate a crucial role of FA elongation and alteration in cardiolipin acyl chain composition in PCSCs, representing attractive therapeutic targets in PDAC.

## Introduction

Pancreatic cancer comprises different types of neoplasia, among which the most common is the infiltrating neoplasm named pancreatic ductal adenocarcinoma (PDAC). PDAC arises in exocrine glands of the pancreas^1,2^ and characterises 85% of pancreatic cancer cases. It represents the eleventh most common cancer worldwide^3^ and the seventh leading cause of cancer-related deaths in the world, being on track to become the second most common cause of cancer-related deaths by 2030^1^. It is the most lethal cancer with a 5-year survival rate of less than 9%^4^. PDAC is defined as an intractable malignancy for several reasons, but mainly due to lack of early diagnosis and effective treatments. Increasing evidence suggests that drug resistance and metastatic capability of PDAC are mainly influenced by the presence of highly plastic stem cells within the tumour, known as pancreatic cancer stem cells (PCSCs). PCSCs, described for the first time in 2007^5^, represent a small (less than 1%) sub-population of undifferentiated quiescent cells^6^ characterized by self-renewal, unique plasticity and metabolism, and capacity to organize the tumour bulk producing a hierarchy of differentiated cells^7^. Currently, the origin of cancer stem cells (CSCs) is still unknown. Two interrelated models have been proposed to explain the heterogeneity, chemoresistance, and aggressiveness of pancreatic cancer^8^. Firstly, the hierarchical model that proposes CSCs as “entities” driving tumour formation, metastasis, chemoresistance, and relapse. Secondly, the stochastic model that describes CSCs as “states”, i.e. they can originate from all cancer cells because of accumulated mutations or epigenetic changes. PCSCs are characterized by aberrant expression of the embryonic stem cell transcription factors, including Oct3/4, Nanog, and Sox2^9,10^, as well as of epithelial-to-mesenchymal transition markers, including CDH1 and Zeb1^6^. Recently, by proteomic and metabolomic profiling, our group reported that PCSCs obtained from Panc-1 PDAC cell line rely on fatty acid and mevalonate pathways for their survival^11^. However, therapies targeting cancer-associated biosynthetic pathways have not given satisfactory results on overall patient survival^12^, since these do not target either the plastic PCSC sub-population or the transient cells that replenish the PCSC pool^8^.

Despite the importance of a multi-omics characterization of PCSC, in-depth characterization is still deficient. In addition to our previous findings^11,13^ few metabolomics^14^ and proteomics^15–17^ studies have been published, while no lipidomic analysis has as yet been carried out on PCSCs. Further elucidation of the signaling pathways that regulate PCSC growth, survival, and metabolomic plasticity is needed to detect therapeutic targets and to explore novel therapeutic approaches against pancreatic cancer^18^. Therefore, in the current study proteomic and lipidomic analyses were integrated to investigate the signaling and metabolic dysfunctions implicated in the pathophysiology of PCSCs obtained from parental (P) PDAC cell lines. Protein and lipid profiles of PCSCs derived from four PDAC cell lines harbouring different genetic backgrounds^19^, e.g. PaCa3 (p16-met), PaCa44 (KRAS^G12V^, p53^C176S^, p16-met), MiaPaCa2 (KRAS^G12C^, p53^R248W^, p16-del), and PC1J (KRAS^G12V^, p53^R175H^, SMAD4/DPC4^D355G^, p16-del) were analysed. To our knowledge, the current work presents the first proteome and lipidome investigation of PaCa3, PaCa44, MiaPaCa2, and PC1J CSCs obtained from relative PDAC cell lines. The results shown here support the potential of a multiomic approach and contribute to the understanding of PDAC biology revealing mitochondrial cardiolipin remodelling, as well as enhanced fatty acid elongation and phosphoinositol phosphatase pathways, as promising targets in PCSCs.

## Results

### PCSCs derived from the four cell lines show different morphological properties

**Figure 1:**
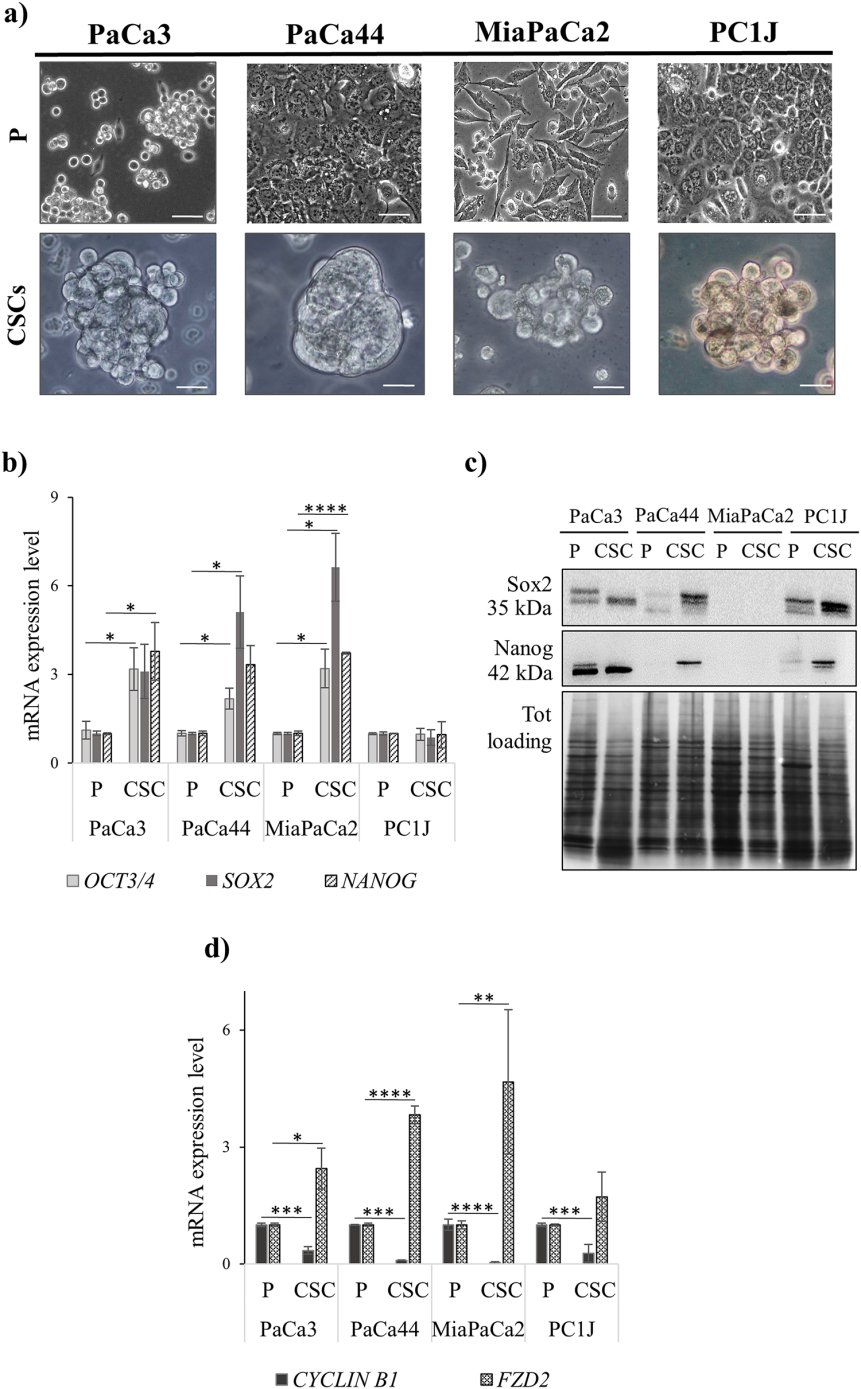
PCSCs grow as spheroids and have dysregulated levels of stemness and quiescence related markers. (**a**) Bright-field microscopy of P cells (upper lane) and PCSCs (bottom lane) obtained after 2 weeks of culture (× 20 magnification). Scale bar = 50 and 25 μm for P cells and PCSCs, respectively. (**b**) Histogram representation of q-PCR analysis of *OCT3/4*, *SOX2*, and *NANOG* gene expressions. (**c**) Immunoblotting assay of Sox2 and Nanog protein expressions. The blots were cropped to focus upon the specific proteins indicated. The full-length blots are presented in Supplementary Figure S1. (**d**) Histogram representation of q-PCR analysis of mRNA expression of *cyclin B1* and *FZD2* gene expressions. Results are reported as means ± standard error (SE) of three independent biological replicates; *p* < 0.05 (*), *p* < 0.01 (**), *p* < 0.001 (***), *p* < 0.0001 (****).

Considering that cancer stem cells grow as spherical formations^20^, the morphological differences between the four PCSCs and P cell lines (PaCa3, PaCa44, MiaPaCa2 and PC1J) were initially investigated. Different growth patterns between P and PCSCs were observed (Fig. 1a), with differences in the shape and size of cells. On one hand, the four P cell lines grew as adherent cells in monolayers showing an epithelial morphology with intact cell-to-cell contacts. On the other hand, PCSCs acquired a mesenchymal morphology forming tumour-spheroids in suspension with features that could depend on the cell line of origin.

### PCSCs’ expression of stem and quiescent markers

mRNA levels of genes related to stemness (i.e. *OCT3/4, SOX2, and NANOG*), cell cycle (*CYCLIN B1*) and Wnt signaling (*FRIZZLED 2, FZD2*) were evaluated. Figure 1b shows that *OCT3/4*, *SOX2* and *NANOG* genes were upregulated in PaCa3, PaCa44, and MiaPaca2 CSCs compared to their respective P cell lines. However, no significant (*p* < 0.05) mRNA level differences of stemness genes were detected for PC1J CSCs. Therefore, protein expression of CSC markers was evaluated by immunoblotting in the four P cells and PCSCs. The results show that Oct3/4 was not detectable in either PCSCs or P cells (data not shown); whilst Sox2 and Nanog were induced in PaCa3, PaCa44, and PC1J CSCs. Moreover, not observable levels were established in MiaPaCa2 for both P and CSC cell lines (Fig. 1c). The mRNA expression of *CYCLIN B1* and *FRIZZLED 2* (*FZD2*) genes was also investigated (Fig. 1d). The results showed a decrease of *CYCLIN B1*, and an increase of *FZD2* in all the four PCSCs compared to P cell lines.

### PCSCs show upregulation of HADHA and dysregulated lipid-metabolism-related pathways

**Figure 2:**
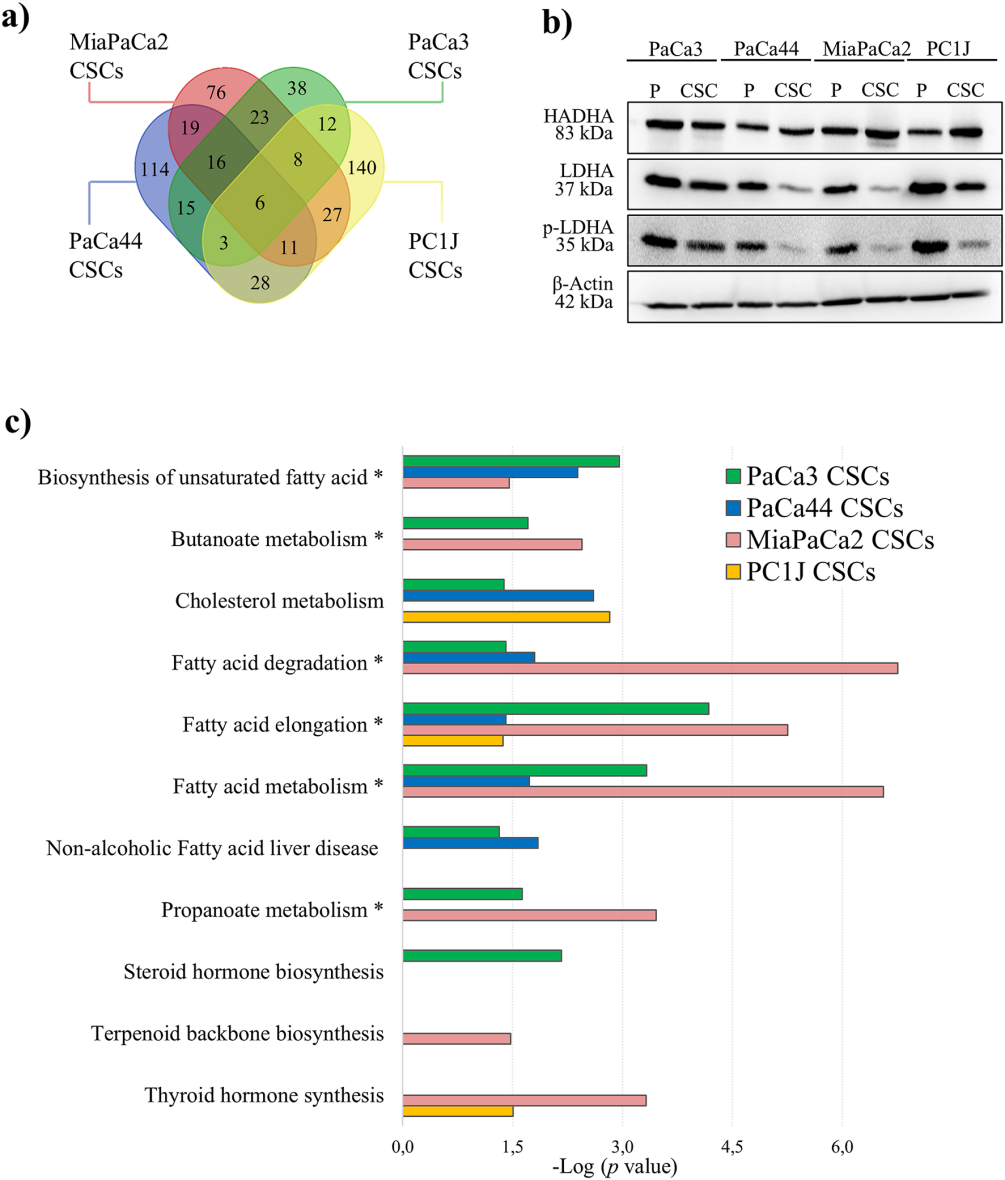
PCSCs display downregulated LDHA, upregulated HADHA, and some specific lipid pathways dysregulated. (**a**) Venn diagram of identified proteins dysregulated in PCSCs in comparison to P cells in the four cell lines (*p* value ≤ 0.05; fold change ± 1.5). (**b**) Immunoblotting assay of HADHA, LDHA and p-LDHA proteins. β-actin was used as a loading control in Western blot analysis. The blots were cropped to focus upon the specific proteins indicated. The full-length blots are presented in Supplementary Figure S1. (**c**) The significantly enriched KEGG pathways (*p* value < 0.05) of the upregulated proteins in PCSCs that are related to lipid metabolism. The asterisk indicates the pathways in which HADHA is involved.

Possible dysregulated signaling and metabolic pathways of PCSCs were investigated by performing proteomics and lipidomics analyses. Comparing the quantitative proteome levels between PCSCs and P cells revealed a total of 121, 186, 212, and 235 proteins differentially expressed in PaCa3, PaCa44, MiaPaCa2, and PC1J CSCs, respectively (Supplementary Table S1). Dysregulated proteins among the different PCSCs were examined and are presented in a Venn diagram in Fig. 2a. Six proteins emerged as dysregulated in all the four PCSCs, where trifunctional enzyme subunit alpha (HADHA, aka hydroxyacyl-CoA dehydrogenase subunit alpha) and voltage-dependent anion-selective channel protein 2 (VDAC2) were upregulated. In contrast, heterogeneous nuclear ribonucleoprotein A/B Isoform 2 (HNRNPAB) and L-lactate dehydrogenase A chain (LDHA) had a lower expression in all the PCSCs compared to P cells. LDHA and HADHA modulation was also monitored by immunoblotting (Fig. 2b). Western blot results indicated that LDHA and p-LDHA were downregulated in PCSCs, whilst HADHA upregulation was detected in MiaPaca2 and PC1J CSCs. Proteomics results revealed that upregulated proteins of PCSCs were mainly mitochondrial proteins and are particularly involved in metabolic pathways and lipid metabolism (Supplementary Table S2). The significantly enriched KEGG pathways (*p* value < 0.05) of upregulated proteins in PCSCs related to lipid metabolism, including FA elongation and biosynthesis of unsaturated FAs, are reported in Fig. 2c. In particular, the pathways in which HADHA is involved are indicated by an asterisk.

### PCSCs display induction of long chain fatty acids and lipid droplets

**Figure 3:**
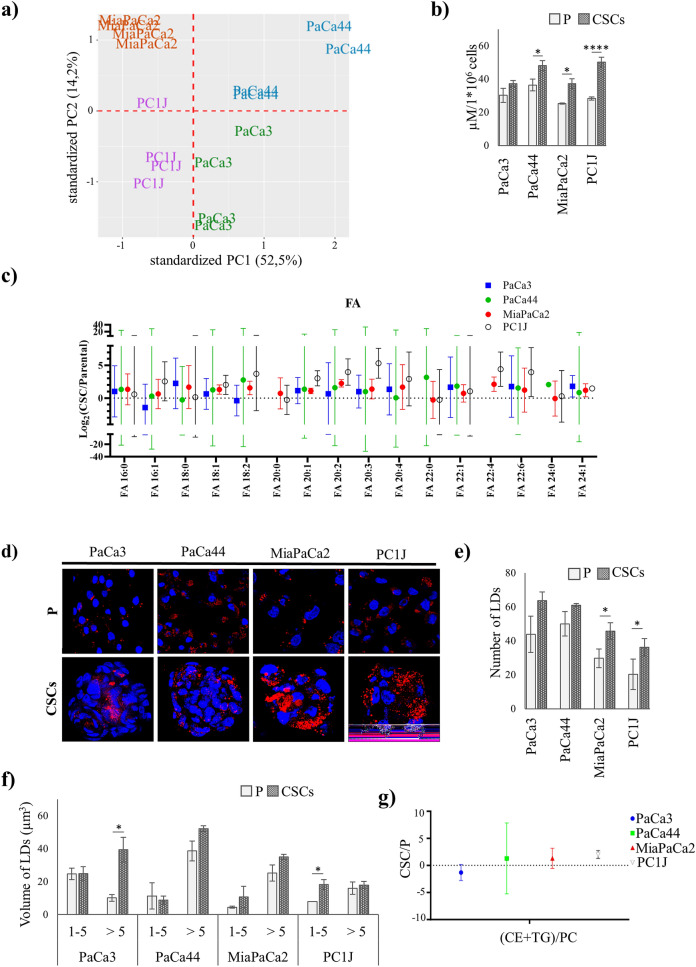
Accumulation of fatty acids and lipid droplets in PCSCs. (**a**) Principal component analysis (PCA) of lipid profile of PaCa3, PaCa44, MiaPaCa2, and PC1J CSCs against P cells. Analysis of FAs as detected by (**b**) FFA Quantification kit and (**c**) LC–MS/MS-based lipidomics (bars show mean fold change ± 95% confidence intervals). (**d**) Representative confocal microscopy images of PCSCs and P cells labelled with Oil Red O (red) to detect LDs, and with DAPI (blue) to highlight nuclei. Measure of (**e**) number and (**f**) size (as volume, μm^3^) of LDs obtained following imaging cells (data are presented as means ± standard error (SE) of three independent experiments); (**g**) analysis of CE + TG content (normalized on PC) detected by LC–MS/MS-based lipidomics (bars show mean fold change ± 95% confidence intervals). *p* < 0.05 (*) and *p* < 0.0001 (****).

The lipid profile of all four pairs of PCSCs and P cells was obtained, and a total of 755 lipid species were quantified. Principal component analysis (PCA) of the fold-change of PCSCs/P ratio showed that all the cell lines cluster away from each other (Fig. 3a) indicating that certain discriminating features are detectable at the lipid level. Lipid subclasses and species dysregulated in PCSCs are all reported in Supplementary Table S3 and Supplementary Figure S2. The results show a general increase in FAs in PCSCs as compared to P cells. Modulation of FA intracellular levels were evaluated in PCSCs and P cells by a colorimetric enzymatic assay, showing the statistically significant (*p* < 0.05) accumulation of FAs in Paca44, MiaPaca2, and PC1J CSCs (Fig. 3b). Although a trend of increase was also detected in Paca3 CSCs. The FA results obtained by LC–MS showed a trend towards increased levels of long (16–20 carbons) and very long (≥ 22 carbons) chain FAs in PCSCs as compared to P cells (Fig. 3c).

LC–MS results also showed an increase of some neutral lipids in PCSCs, therefore cells were imaged using confocal microscopy to evaluate lipid droplets (LDs) properties (Fig. 3d). A trend of induction, in either number (Fig. 3e) or volume (Fig. 3f) of LDs was detected in all the four PCSCs. Lipidomic analysis showed a trend of increased content of two major lipids of LDs, TG and CE, in all the PCSCs except for Paca3 CSCs (Fig. 3g) which, however, seems to be characterized by a greater number of LDs.

### Phosphoinositide signaling and fatty acid elongation are peculiar pathways of PCSCs

**Figure 4:**
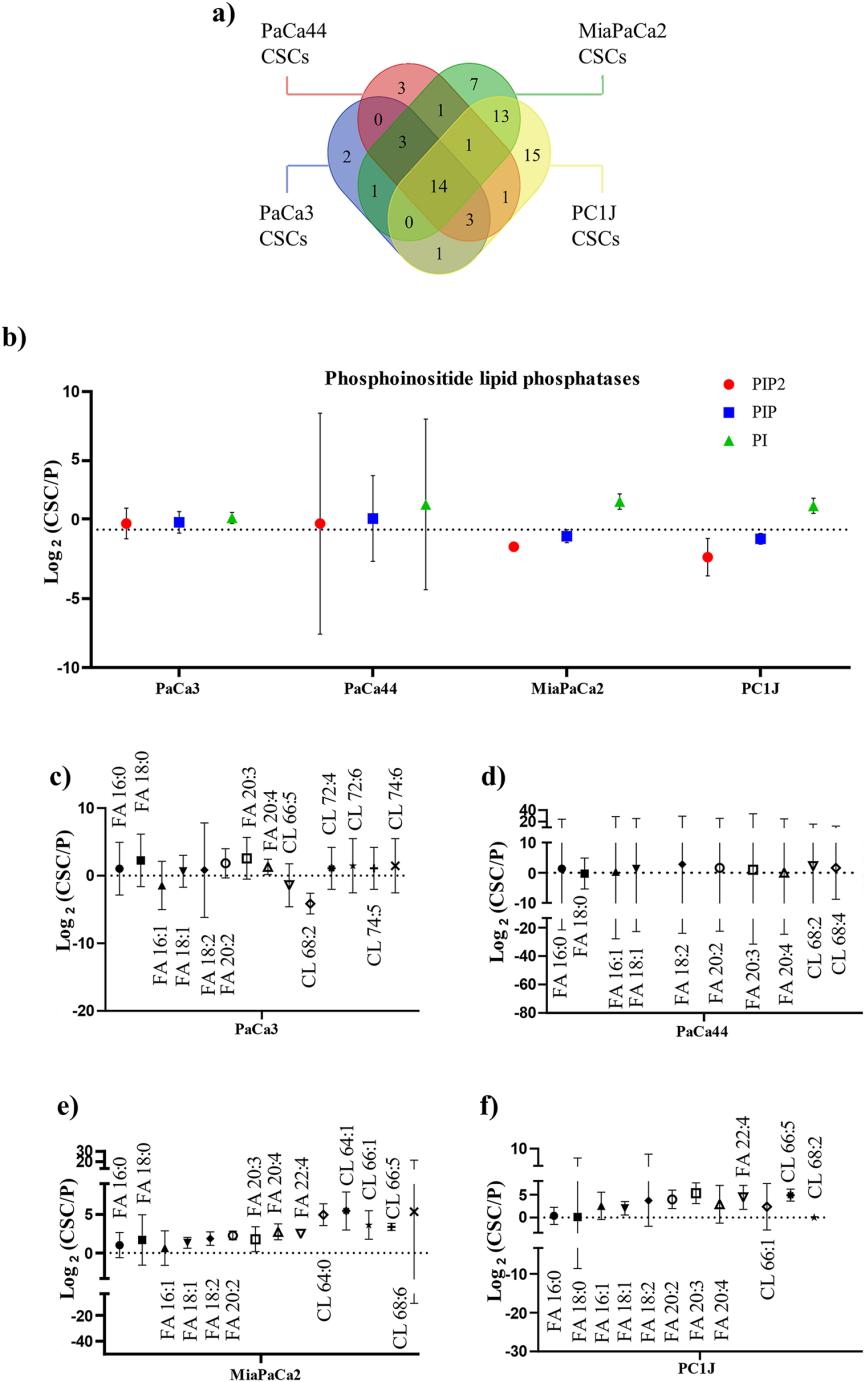
Increased levels of phosphatidylinositol, fatty acids and cardiolipin molecular species characterize PCSCs. (**a**) Venn diagram representation of genes implicate in active lipid pathways found using BioPAN. (**b**) Log_2_ ratio (PCSC/P) in the four PDAC cell lines of the values of phosphatidylinositol diphosphate (PIP2), phosphatidylinositol monophosphate (PIP), and phosphatidylinositol (PI). Log_2_ ratio (PCSC/P) in (**c**) PaCa3, (**d**) PaCa44, (**e**) MiaPaCa2, and (**f**) PC1J cells of the FA substrates and products catalysed by Elovl5, including FA 16:0 and CL molecular species remodelled by HADHA enzyme. Bars show mean fold change ± 95% confidence intervals.

The lipidomics analysis performed by BioPAN revealed active pathways and related genes for each PCSCs (Table 1). BioPAN combines current knowledge of lipid metabolism with a statistical analysis framework, by comparing two biological conditions to identify activated or suppressed pathways, and presents the results in an interactive graphical display, ranking the reactions using Z-score values. Each gene activates or supresses enzymes catalysing lipid metabolic pathways (Supplementary Figure S3), and a Venn diagram representation was used to show overlapping between these genes (Fig. 4a). The results highlighted 14 common genes involved in pathways predicted as active in all four PCSCs compared to P cells. Among these, thirteen (i.e., *SYNJ1, SYNJ2, SACM1L, MTMR1, MTMR2, MTMR3, MTMR4, MTMR6, MTMR7, MTMR8, MTMR9, MTMR14,* and *PTEN*) encode for phosphoinositide lipid phosphatase proteins, and one for the fatty acid elongase-5 (*ELOVL5*).

**Table 1:** BioPAN predicted significantly active reactions chains (Z > 1.645) of lipid class and FA species and relative genes for each reaction of PCSC lines.

Reaction chain	*Z*-score	Predicted genes
**PaCa3 CSCs**		
SM → Cer	3.014	*SMPD2*, *SMPD3*
O-DG → O-PE → P-PE	2.612	*CEPT1*, *TMEM189*
PIP2 → PIP → PI	2.211	*FIG4*, *OCRL*, *INPP5E*, *PTEN*, *SYNJ1*, *SYNJ2*, *SACM1L*, *MTMR1*, *MTMR2*, *MTMR3*, *MTMR4*, *MTMR6*, *MTMR7*, *MTMR8*, *MTMR9*, *MTMR14*, *PTEN*
O-DG → O-PC	2.074	*CHPT1*
FA 16:1 → FA 18:1 → FA 20:1 → FA 22:1 → FA 24:1	1.834	*ELOVL5*, *ELOVL6*, *ELOVL3*, *ELOVL3*, *ELOVL3*
dhSM → dhCer	1.828	*SGMS1*, *SGMS2*
DG → PE	1.673	*CEPT1*
PC → PS	1.654	*PTDSS1*
**PaCa44 ﻿CSCs﻿**		
FA 16:1 → FA 18:1 → FA 18:2	2.721	*ELOVL5*, *ELOVL6*, *FADS2*
O-PE → P-PE	2.620	*TMEM189*
dhCer → Cer → SM	2.221	*DEGS1*, *DEGS2*, *SGMS1*, *SGMS2*, *CERT1*
O-DG → O-PC	2.022	*CHPT1*
FA 18:1 → FA 18:2	1.930	*FADS2*
dhCer → dhSM	1.919	*SGMS1*, *SGMS2*
O-DG → O-PE → P-PE → P-PC	1.895	*CEPT1*, *TMEM189*, *PLD1*
DG → PE	1.859	*CEPT1*
PS → PE	1.805	*PISD*
DG → PC	1.685	*CHPT1*
PIP → PI	1.667	*SYNJ1*, *SYNJ2*, *SACM1L*, *MTMR1*, *MTMR2*, *MTMR3*, *MTMR4*, *MTMR6*, *MTMR7*, *MTMR8*, *MTMR9*, *MTMR14*, *PTEN*
**MiaPaCa2 ﻿CSCs﻿**		
PIP2 → PIP → PI	3.731	*FIG4*, *OCRL*, *INPP5E*, *PTEN*, *SYNJ1*, *SYNJ2*, *SACM1L*, *MTMR1*, *MTMR2*, *MTMR3*, *MTMR4*, *MTMR6*, *MTMR7*, *MTMR8*, *MTMR9*, *MTMR14*, *PTEN*
PIP → PI	2.392	*SYNJ1*, *SYNJ2*, *SACM1L*, *MTMR1*, *MTMR2*, *MTMR3*, *MTMR4*, *MTMR6*, *MTMR7*, *MTMR8*, *MTMR9*, *MTMR14*, *PTEN*
dhCer → dhSM	2.103	*SGMS1*, *SGMS2*
Cer → SM	2.049	*SGMS1*, *SGMS2*, *CERT1*
PIP2 → DG	2.007	*PLCB1*, *PLCB2*, *PLCB3*, *PLCB4*, *PLCD1*, *PLCD3*, *PLCD4*, *PLCE1*, *PLCG1*, *PLCG2*
PA → DG	1.983	*PLPP1*, *PLPP2*, *PLPP3*
O-LPE → O-LPA	1.944	*PLD1*
PE → PC	1.941	*PEMT*
PG → CL	1.821	*CRLS1*
O-PE → P-PE → P-PC	1.811	*TMEM189*, *PLD1*
PA → PS	1.782	*CDS1*, *PTDSS1*
PE → PS	1.779	*PTDSS2*
FA 18:2 → FA 20:2	1.717	*ELOVL5*
**PC1J ﻿CSCs﻿**		
O-DG → O-PC	3.644	*CHPT1*
PIP2 → PIP → PI	3.129	*FIG4*, *OCRL*, *INPP5E*, *PTEN*, *SYNJ1*, *SYNJ2*, *SACM1L*, *MTMR1*, *MTMR2*, *MTMR3*, *MTMR4*, *MTMR6*, *MTMR7*, *MTMR8*, *MTMR9*, *MTMR14*, *PTEN*
PIP2 → DG → PE	2.879	*PLCB1*, *PLCB2*, *PLCB3*, *PLCB4*, *PLCD1*, *PLCD3*, *PLCD4*, *PLCE1*, *PLCG1*, *PLCG2*, *CEPT1*
FA 16:0 → FA 16:1 → FA 18:1 → FA 20:1	2.527	*SCD3*, *ELOVL5*, *ELOVL6*, *ELOVL3*
PIP2 → DG → PC → PA	2.378	*PLCB1*, *PLCB2*, *PLCB3*, *PLCB4*, *PLCD1*, *PLCD3*, *PLCD4*, *PLCE1*, *PLCG1*, *PLCG2*, *CHPT1*, *PLD1*, *PLD2*
FA 18:0 → FA 18:1 → FA 18:2 → FA 20:2 → FA 20:3 → FA 20:4	2.349	*SCD1*, *FADS2*, *ELOVL5*, *FADS1*, *FADS1*
DG → PC	2.343	*CHPT1*
PIP2 → DG → PA	2.265	*PLCB1*, *PLCB2*, *PLCB3*, *PLCB4*, *PLCD1*, *PLCD3*, *PLCD4*, *PLCE1*, *PLCG1*, *PLCG2*, *DGKA*, *DGKB*, *DGKD*, *DGKE*, *DGKG*, *DGKH*, *DGKI*, *DGKK*, *DGKQ*, *DGKZ*
PIP → PI	2.148	*SYNJ1*, *SYNJ2*, *SACM1L*, *MTMR1*, *MTMR2*, *MTMR3*, *MTMR4*, *MTMR6*, *MTMR7*, *MTMR8*, *MTMR9*, *MTMR14*, *PTEN*
FA 18:0 → FA 18:1 → FA 20:1 → FA 22:1	2.098	*SCD1*, *ELOVL3*, *ELOVL3*
DG → PE	2.022	*CEPT1*
FA 16:0 → FA 16:1 → FA 18:1 → FA 18:2 → FA 20:2 → FA 20:3 → FA 20:4	1.982	*SCD3*, *ELOVL5*, *ELOVL6*, *FADS2*, *ELOVL5*, *FADS1*, *FADS1*
O-DG → O-PE	1.949	*CEPT1*
PIP2 → DG → PC → CL	1.931	*PLCB1*, *PLCB2*, *PLCB3*, *PLCB4*, *PLCD1*, *PLCD3*, *PLCD4*, *PLCE1*, *PLCG1*, *PLCG2*, *CHPT1*, *TAZ*
FA 20:2 → FA 20:3	1.926	*FADS1*

As concerning the substrates and the products of phosphoinositide lipid phosphatases, a statistically significant increase (at 95% CI) in the levels of phosphatidylinositol (PI) in PaCa3, MiaPaCa2 and PC1J CSCs was observed (Fig. 4b). In addition, the levels of phosphatidylinositol monophosphate (PIP) appeared to be not statistically significant (at 95% CI) changed, whilst the levels of phosphatidylinositol diphosphate (PIP2) showed a 1.2 and two-fold statistically significant decrease in MiaPaCa2 and PC1J CSCs.

Based on the proteomics results, FA elongation and biosynthesis of unsaturated FAs emerged as key pathways for PCSCs, therefore the role of the elongases and desaturases was also investigated in the FA metabolism (Supplementary Figure S4). Genes associated with the biosynthesis of FAs, among which *ELOVL5*, are also included in Table 1. As concerning products of the catalytic activity of Elovl5, a general increase in long and very long-chain unsaturated FAs was detected in PCSCs, with the exception of PaCa44 CSCs (Fig. 4c–f). In particular, statistically significant (at 95% CI) increased levels of FAs 18:1, 20:2, 20:3, 22:4 were detected in MiaPaca2 and PC1J CSCs, and of FA 20:4 in Paca3, MiaPaca2 CSCs.

### PCSCs are characterized by alteration in cardiolipin acyl chain composition

CL molecular species, which could contain fatty acyl chain incorporation of C18:1, C18:2, and C16:0 are shown in Fig. 4c–f. A trend of increase was observed in some CL molecular species of PCSCs. On the contrary, a statistically significant decrease (95% CI) of 4.2-fold was only observed in CL 68:2 of PaCa3 CSCs (Fig. 4a). Interestingly, a statistically significant (95% CI) increase was observed in CL 64:0 (fivefold), CL 64:1 (5.5-fold), CL 66:1 (3.5-fold), and CL 66:5 (3.2-fold) in MiaPaCa2 CSCs (Fig. 4c). In addition, a fivefold a statistically significant increment in CL 66:5 molecular species were detected in PC1J CSCs (Fig. 4d).

## Discussion

PCSCs play a crucial role in PDAC initiation and metastasis and are responsible for resistance to chemotherapy and radiation, however these cells are still not completely characterized from a molecular point of view. Here we find that Sox2, Oct3/4 and Nanog are induced in PCSCs when compared to P cells. These transcriptional factors are key players in inducing stemness in cancer cells^21^. Strangely, we detected increased Sox2 and Nanog expression at the protein level, but not at the mRNA level, in PC1J CSCs, which may be explained by different half-life and/or degradation rate of mRNAs in these cells as compared to the other PDAC cell lines. On the contrary, increased Sox2, Oct3/4 and Nanog expression at mRNA level, but not at the protein level, was detected in MiaPaca2 CSCs suggesting that stem cell markers of these cells may have undetectable expression under the experimental conditions or a modified epitope which preclude their immunodetection. Our results also show that PCSCs have reduced expression of *cyclinB1* and induced expression *FZD2* at mRNA level. Cyclin B1 plays a key role in the transition from the G2 to M phase of the mitotic process, suggesting a G2/M phase arrest of analysed PCSCs. Accordingly, it has been reported that some CSCs are slow-cycling quiescent cells^22^, thus not dividing while retaining the ability to re-enter cell proliferation. Whilst the observed increase in *FZD2* is in agreement with previously reported results that show Fzd2 involvement in the activation of Wnt signaling, a key regulating gene in CSCs^23^ as well as in drug resistance^24^. Moreover, Fzd2 has been proposed as a novel target for molecular therapy of pancreatic cancer^25^.

Our comprehensive analysis of protein modulation in PCSCs and P cells portrays specific and common proteome changes for the four PCSCs, most of which are related to dysregulated lipid-metabolism pathways. Interestingly, among these latter are the biosynthesis and metabolism of cholesterol, the main product of the already reported mevalonate pathway^11^. Currently, the debate on PCSC metabolism is still open, as some studies revealed PCSCs to be highly glycolysis-dependent^6^ while others point to oxidative phosphorylation (OXPHOS) dependency^26^. LDHA was found downregulated in all the PCSCs, which is consistent with the OXPHOS as energy source in PCSCs rather than glycolysis^27^. Moreover, LDHA phosphorylation of tyrosine in position 10 was also evaluated by immunoblotting, as p-LDHA promotes the activity of LDHA and the Warburg effect^28^, demonstrating a downregulation of p-LDHA in PCSCs.

To further dissect the metabolic pathways, a comprehensive analysis of lipid modulations in PCSCs and P cells was performed. The findings suggest that PCSCs are characterized by induction of long chain fatty acids and accumulation of lipid droplets. Phosphoinositide signaling and fatty acid elongation come off as specifically noteworthy pathways in PCSCs. Accordingly, some main genes involved in these pathways were detected: *PTEN* (among many other phosphoinositide lipid phosphatases) and *ELOVL5*. Pten has a dual-specificity phosphatase activity: protein and lipid dephosphorylation. As a lipid phosphatase, preferentially dephosphorylates phosphoinositide substrates, removing the phosphate from phosphatidylinositol 3,4,5-trisphosphate (PIP3), PIP2, PIP and inositol 1,3,4,5-tetrakisphosphate (I4P)^29,30^. In contrast, Elovl5 plays a role in the addition of two carbon units to the carboxyl ends of fatty acyl CoA substrates, acting on monounsaturated palmitoleoyl-CoA (C16:1)^31^ and linoleoyl-CoA (C18:2), and participating in the production of very long chain FAs 20:3, 20:4, 22:4 and 22:5^32,33^.

Interestingly, lipid biosynthetic pathway analysis did not show genes in reaction pathways involving Cardiolipin (CL) as no increased levels were detected; despite the fact that proteomics showed an upregulation of HADHA in all the four PCSCs, and a significant enrichment of “acyl chain remodelling of CL” pathway for CSCs derived from MiaPaca2 and PC1J cells. CL is a major mitochondrial membrane glycerophospholipid subclass^34^ and comprises two diacylglycerol phosphate residues connected by a glycerol backbone and four fatty acyl chains. CL is derived from the catalytic activity of Cardiolipin synthase (CRLS) on two units of phosphatidylglycerol (PG), but activity of the phospholipase A2 produces monolysocardiolipin, which is the substrate for HADHA. Therefore, fatty acyl chain combinations in CL give a wide variety of mature CL species. On the one hand HADHA catalyses the last three steps of mitochondrial β-oxidation of FAs, when is accompanied by the β subunit (HADB)^35^. On the other hand, HADHA plays a role in the acylation of monolysocardiolipin to form CL^36,37^. Thus, further analysis of the CL lipid molecular species linked with CL remodelling by HADHA were performed. Our lipidomic analysis indicated, for MiaPaCa2 and PC1J CSCs, increased levels of CL molecular species showing incorporation of oleoyl-CoA (C18:1), linoleoyl-CoA (C18:2), and palmitoyl-CoA (C16:0). However, not direct correlation can be suggested with CL results and HADHA upregulation.

It has been reported that changes in CL molecular species composition has an enormous impact on the structural integrity of the inner membrane of mitochondria and on the enzymatic activity of all mitochondrial respiratory complexes^38,39^. These protein complexes can be linked with oxidative stress modulation by reduction of superoxide levels, proton pump homeostasis, and energy metabolism^40^. Therefore, CL plays a critical role in oxidative phosphorylation (OXPHOS). During this process, many protons are transported across the mitochondrial membrane, causing a pH shift. In this context, CL molecular species function as a proton trap within the membranes of this organelle^41^. Mechanistically, H^+^ ions interact with PO4^−^ groups of CL to neutralize its negative charge and to reduce the size of the hydrate coat around the polar head^42^. The neutralized CL acquires a reverse molecular shape, a non-bilayer packing of CL results, and this leads to inverted micelles accumulating near highly-protonated regions of membranes. These non-bilayer structures favour the oligomerization of the F0 sector of the ATP synthase complex and act as a proton trap to shuttle protons to the ATP synthase complex leading to enhanced energy production^42,43^. Very little recently published research have shown a prominent role for CL alterations in cancer stem cells. Interestingly, it has been shown that inhibition of mitochondrial phospholipid production, such as CL, reduces stemness and increases differentiation of acute myeloid leukaemia cells^44,45^. To the best of our knowledge, this is the first time that the changes in CL acyl-chain composition have been demonstrated for a solid tumour like PDAC as well as its PCSCs.

In conclusion, a proteomic and lipidomic analysis of pancreatic parental cancer cells and cancer stem cells established major differences in the signaling and lipid metabolic pathways between these two cell types. In summary, the data obtained in this multiomics study showed that alteration of cardiolipin acyl-chain composition, fatty acid elongation, and phosphoinositide signaling are specific pathways of PCSCs and potential new targets in novel therapeutic strategies for pancreatic cancer.

## Materials and methods

### Cell culture

The human PDAC cell lines PaCa3, PaCa44, MiaPaCa2, and PC1J, called parental (P) cells, were obtained from American Type Culture Collection (ATCC). These cells were grown in RPMI 1640 media supplemented with 10% foetal bovine serum, 2 mM glutamine, and 50 µg/ml gentamicin sulphate (Gibco, Life Technologies). Cells were maintained at 37 °C with 5% CO_2_ for some passages. As previously described by Dalla Pozza et al.^46^, PCSCs were obtained by growing adherent cells in “CSC medium” (i.e. DMEM/F‑12, B27, 1 g/l glucose, fungizone, penicillin/streptomycin, heparin, epidermal growth factor and fibroblast growth factor) for 2 weeks. “CSC medium” was refreshed twice a week with new medium solution and maintained at 37 °C with 5% CO_2_. PCSCs were passed through a cell strainer (> 40 μm) to separate cell spheres, followed by examination under a light microscope (Axio Vert. A1, Zeiss) at 20 × and 40 × magnifications, prior to spheres collection. The cell numbers and viabilities of P cells and PCSCs were determined with the trypan blue exclusion test. Cells with viability higher than 85% were pelleted, frozen with liquid nitrogen, and stored at − 80 °C for further analyses.

### RNA extraction and qPCR

Total RNA was extracted from 1 × 10^6^ cells (PaCa3, PaCa44, MiaPaCa2, and PC1J parental and stem cells) using TRIzol Reagent (Life Technologies) according to the manufacturer's instructions. The extracted RNA was quantified by NanoDrop One (Thermo Fisher Scientific) and checked for integrity loading on 1.5% agarose gel. 1 μg of RNA was reverse transcribed using first-strand cDNA synthesis. The real-time PCR reaction was performed according to the protocol of the SYBR‑Green detection chemistry with GoTaq qPCR Master Mix (Promega) on a QuantStudio 3 Real-Time PCR System (Thermo Fisher Scientific). The sequence of the primers used in this experiment are provided in Supplementary Table S4. The cycling conditions used were: 95 °C for 10 min, 40 cycles at 95 °C for 15 s, 60 °C for 1 min, 95 °C for 15 s, and 60 °C for 15 s. Reactions were run in triplicate in three independent biological experiments. Expression data were normalized on the housekeeping SDHA and were analysed using the 2^−ΔΔCT^ method.

### Protein extraction and LC–MS/MS proteomics analysis

Identification and quantification of proteome modulation of P cells and CSCs of the four cell lines were performed as previously reported by Brandi et al.^47^. Briefly, cells were collected (three biological replicates for each cell type), washed and lysed in 1X PBS with protease inhibitors cocktail and 0.1% SDS. Acetone was used for protein precipitation/denaturation followed by resuspension in 100 mM NH_4_HCO_3_. Protein content was measured by Bicinchoninic Acid Protein Assay. Thirty µg of protein extract was subjected to reduction (with dithiothreitol) and alkylation (with iodoacetamide), prior to tryptic digestion at 37 °C overnight.

Tryptic peptides were analysed by label-free LC–MS/MS as previously^48^, performed by using a micro-LC system (Eksigent Technologies, Dublin, USA) interfaced with a 5600 + TripleTOF mass spectrometer (AB SCIEX, Concord, Canada). Samples were subjected first to data-dependent acquisition (DDA) analysis to generate the SWATH-MS spectral library, and then to cyclic data independent analysis (DIA), based on a 25-Da window, using three technical replicates of each sample. The MS data were acquired by Analyst TF v.1.7 (AB SCIEX), while PeakView v.1.2.0.3, Protein Pilot v.4.2 (AB SCIEX) and Mascot v.2.4 (Matrix Science) programs were used to generate the peak-list. The database search was performed using the UniProt/Swissprot (v.2018.01.02, 42271 sequences entries). Samples were input in the Protein Pilot software v. 4.2 (AB SCIEX, Concord, Canada), with the following parameters: cysteine alkylation, digestion by trypsin, no special factors and FDR at 1% were used for database search with Protein Pilot, while for Mascot search the following parameters were used: trypsin as digestion enzyme, 2 missed cleavages, search tolerance of 50 ppm for the peptide mass tolerance and 0.1 Da for the MS/MS tolerance. The charges of the peptides to search for were set to 2 +, 3 + and 4 +, and the search was set on monoisotopic mass. The instrument was set to ESI-QUAD-TOF and the following modifications were specified for the search: carbamidomethyl cysteines as fixed modification and oxidized methionine as variable modification. FDR was fixed at 1%. Peak lists generated by both Protein Pilot and Mascot were compared to UniProt/Swissprot (v.2018.01.02, 42271 sequences entries) database. The obtained files from the DDA acquisitions were used for the library generation using a FDR threshold of 1%. Protein quantification was performed by PeakView v.2.0 and MarkerView v.1.2. (AB SCIEX) programs by extracting from SWATH files ten peptides per protein with the highest MS1 intensity, and ten transitions per peptide. Peptides with FDR lower than 1.0% were exported, and up- and downregulated proteins were selected using *p* value < 0.05 and fold change > 1.5.

### Lipid extraction and LC–MS/MS lipidomics analysis

Lipids extraction and analysis was performed as previously outlined^49^. Briefly, frozen cell pellet lipids were extracted using the Folch method with Chloroform/Methanol/Water (2:1:1 ratio). Lipids were dried using a SpeedVac (Savant SP131DDA, Thermo Scientific, Runcorn, UK) and re-suspended in Chloroform/Methanol (1:1), prior to injection into a Shimadzu Prominence 20-AD system (Shimadzu, Kyoto, Japan). Chromatographic separation was achieved using a Waters Acquity UPLC C4 (100 × 1 mm, 1.7 μm particle size) column (Milford, MA, U.S.A.). The column was kept a 45 °C and 7 μl of samples were eluted using a mobile phase composed of solvent A (water) and B (acetonitrile), each containing 0.025% formic acid. The gradient started at 45% B for 5 min, then increased to 90% B for 5 min, and 100% B was reached after an additional 10 min and held for 7 min before re-equilibration at 45% B for 5 min. The flow rate was maintained at 100 μl/min. Accurate mass (with an error below 5 ppm) was acquired on an Orbitrap Elite mass spectrometer (Thermo Fisher Scientific, Waltham, MA, USA). Source parameters for positive polarity were: capillary temperature 275 °C; source heater temperature 200 °C; sheath gas 10 AU; aux gas 5 AU; sweep gas 5 AU. Source voltage was 3.8 kV. Full scan spectra in the range of m/z 340–1500 were acquired at a target resolution of 240,000 (FWHM at m/z 400). Manual inspection of the results was carried out using Xcalibur and further processed using Lipid Data Analyzer (LDA) 2.7.0_2019 software^50^.

Targeted analysis of lysolipids, Cer, and dhCer, was performed using a QTRAP 6500 LC–MS/MS System (AB SCIEX) operating in MRM mode. Quantification of multiple species of ceramides was carried out by the integration of the peak area as normalized against the peak area of the non-endogenous odd-chain ceramide C17:0. C17 was present at a known concentration and served as the internal standard (IS). Collision energy (CE) was optimized previously.

Thirtyfive lipid subclasses were identified, including alkenyl-acylphosphatidylcholine (P-PC), alkenyl-acylphosphatidylethanolamine (P-PE), alkyl-acylglycerol (O-DG), alkyl-acylphosphatidylcholine (O-PC), alkyl-acylphosphatidylethanolamine (O-PE), alkyl-triacylglycerol (O-TG), cardiolipin (CL), ceramides (Cer), cholesterol (CH), cholesterol ester (CE), diacylglycerol (DG), dihydroceramides (dhCer), dihydrosphingomyelin (dhSM), free fatty acids (FA), phosphatidic acid (PA), phosphatidylcholine (PC), phosphatidylethanolamine (PE), phosphatidylglycerol (PG), phosphatidylinositol (PI), phosphatidylserine (PS), sphingomyelin (SM), sphingosine (SG), triacylglycerol (TG), lysophosphatidic acid (LPA), lysophosphatidylcholine (LPC), lysophosphatidylethanolamine (LPE), lysophosphatidylglycerol (LPG), lyso phosphatidylinositol (LPI), phosphatidylserine (LPS), alkenyl-lysophosphatidylcholine (P-LPC), alkenyl-lysophosphatidic acid (P-LPA), alkyl-lysophosphatidylethanolamine (O-LPE), alkyl-lysolysophosphatidylcholine (O-LPC), phosphatidylinositolmonophosphate (PIP), phosphatidylinositoldiphosphate (PIP2), phosphatidylinositoltriphosphate (PIP3).

Lipid relative quantitation levels were calculated using the R-studio (v3.2.4) software^51^ (https://www.R-project.org) with in-house built scripts. Statistical comparison between the P cells and PCSCs was performed using the paired *t-*test (*p* < 0.005), principal component analysis (PCA), and log_2_ ratio transformation of PCSCs versus parental lipid levels.

### Bioinformatics analysis of omics data

Bioinformatics analysis of proteomic data was performed as previously described^52^. Briefly, to characterize the function of proteins, gene ontology (GO) annotation, KEGG and Reactome pathways enrichment analyses were performed using STRING v.11.0 (http://string-db.org)^53^. The differentially expressed proteins were analysed for candidate functions and pathways enrichment, setting Homo sapiens as taxonomy, *p* < 0.05 and gene count > 2 as cut-off point.

Lipid profile levels obtained from the four PCSCs and P cells were loaded into an open access tool BioPAN, on LIPID MAPS Lipidomics Gateway (https://lipidmaps.org/biopan/)^54^. BioPAN provides Z-scores (Z > 1.645 at *p* < 0.05) using substrate and product lipid levels and integrate a list of genes, which could be involved in the activation or suppression of enzymes catalysing lipid metabolic pathways.

### Western blot analysis

Western Blot analysis was performed as previously described^55^. Briefly, proteins were resolved on 12% SDS–PAGE gels and transferred to PVDF membrane. After transferring proteins onto PVDF membranes, Amido Black 1X Staining Solution was used to confirm equal protein loading in different lanes. The membranes were blocked with 5% non-fat dry milk in 0.1% Tris-buffered saline (TBS)/Tween-20 and incubated with primary antibodies diluted as reported in Supplementary Table S5, at 4 °C overnight. Once incubation with secondary antibodies was carried out, detection by enhanced chemiluminescence was performed. The chemiluminescent signal was acquired through a ChemiDoc MP Imager (Bio-Rad) using the Image Lab 5.2.1 software (Bio-Rad).

### Confocal fluorescence microscopy

CSCs and P cells were grown on a L-lysine coverslip inside a 24-well plate and incubated at 37 °C (5% CO_2_) for lipid droplets (LDs) staining. Supernatant was removed and, after washing with PBS, cells were fixed using 8% formaldehyde, for 10 min at room temperature. After fixation and washing, cells were incubated with Oil Red O (Bio-Optica) for 20 min at room temperature. Thus, cells were washed again, treated with 0.1% Triton X-100 and 1% bovine serum albumin for 15 min at room temperature, and stained with DAPI (4′,6-diamidino-2-phenylindole nuclei stain, dilution 1:1000; Sigma Aldrich) for 30 min for nuclei visualization. Samples were scanned by a confocal microscope (Leica TCS SP5 AOBS) using 416 nm and 545 nm lasers under a 40 × oil objective. The images were analysed using Leica Application Suite X 3.7.0.20979 software. Imaris X64 9.1.0 software to generate z- and three-dimensional projections for the analysis of lipid LDs shape and volume. Twenty images per sample were recorded and LDs were counted manually using the ImageJ multipoint tool after background correction. Detected LDs were normalized per cell nucleus. Statistical analysis (*t*-test) was performed to identify significant changes.

### Assessment of free fatty acids levels

The levels of free fatty acid were determined using a FFA Quantification Kit (MAK044, Sigma Aldrich) according to the manufacturer's instructions. Briefly, cells were lysed in 1% Triton X-100 in chloroform (w/v) and centrifuged at 13,000 × g for 10 min to remove insoluble debris. The organic phase was collected, dried on 50 °C dry bath for 20 min, fatty acid assay buffer was added and then dissolved by extensive vortex mixing. After that, absorbances for individual wells were read at 570 nm using a microplate reader, and values reported as μM FFA/million cells.

## Supplementary Information

Supplementary Information 1.

Supplementary Information 2.

Supplementary Information 3.

Supplementary Information 4.

Supplementary Information 5.

Supplementary Information 6.

Supplementary Information 7.

## Data Availability

Data are available via ProteomeXchange with identifier PXD023069, username: reviewer_pxd023069@ebi.ac.uk, password: HE4R6eQ3.

## Abbreviations

PDAC: Pancreatic ductal adenocarcinoma
PCSCs: Pancreatic cancer stem cells
CSCs: Cancer stem cells
HADHA: Hydroxyacyl-CoA dehydrogenase subunit alpha, trifunctional enzyme
CL: Cardiolipin
FA: Fatty acid
