# Supplementary material for: Integrated lipidomics and proteomics reveal cardiolipin alterations, upregulation of HADHA and long chain fatty acids in pancreatic cancer stem cells
Source: Sci Rep. 2021 Jun 24;11:13297. doi: 10.1038/s41598-021-92752-5 (PMC8225828; doi:10.1038/s41598-021-92752-5)
Supplement: Supplementary file 1 — Supplementary Information 1. [file 41598_2021_92752_MOESM1_ESM.docx]

# Integrated lipidomics and proteomics reveal cardiolipin alterations, upregulation of HADHA and long chain fatty acids in pancreatic cancer stem cells

Claudia Di Carlo^1^, Bebiana C. Sousa^2^, Marcello Manfredi^3^, Jessica Brandi^1^, Elisa Dalla Pozza^4^, Emilio Marengo^3^, Marta Palmieri^4^, Ilaria Dando^4^, Michael J.O. Wakelam^2^, Andrea F. Lopez-Clavijo^2^, Daniela Cecconi^1^*

## **Supplementary Information**

Supplementary Figure S1-S4


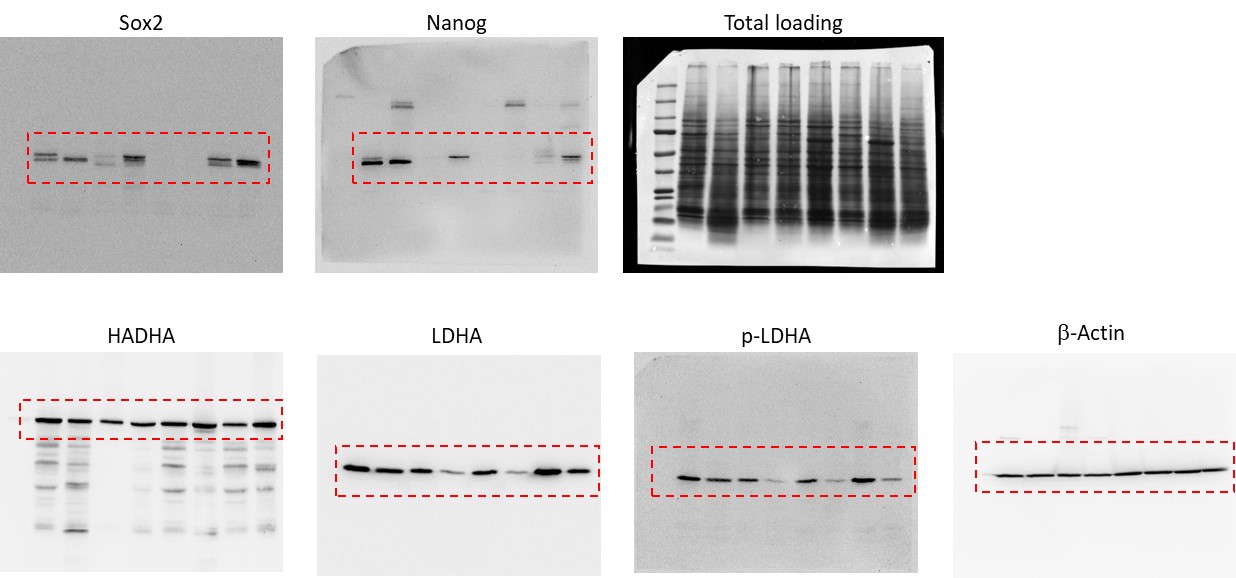


**Supplementary Figure S1.**

Uncropped images and cropped area of western blots.

**Supplementary Figure S2.**

Log_2_ of the fold changes of the lipidome profile of PaCa3 (blue), MiaPaCa2 (red), PaCa44 (green), and PC1J (white) CSCs compared to related P cells. The x-axis represents the 37 lipid subclasses; the y-axis reports the log_2_ ratio (CSC/P) at 95% CI.


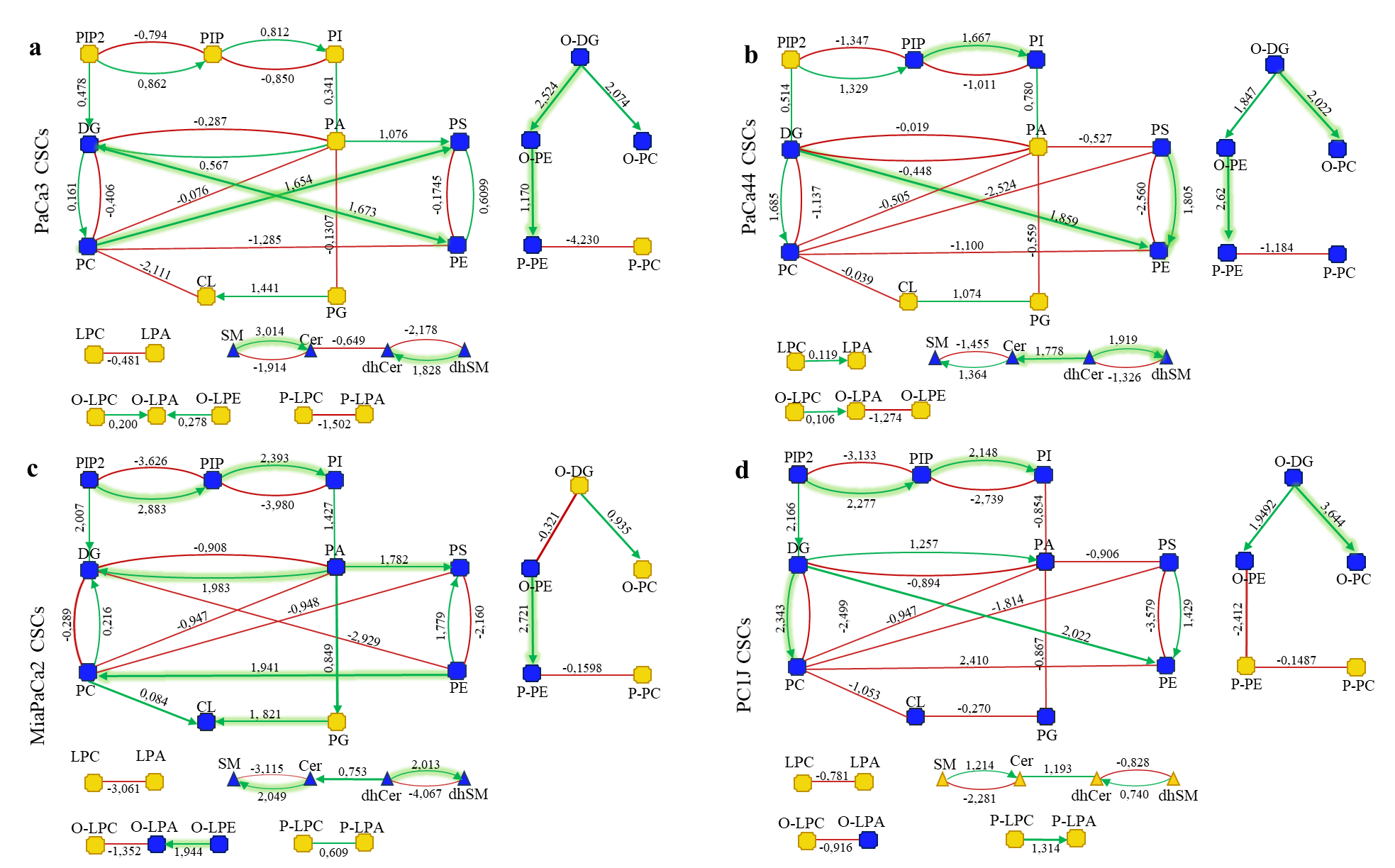


**Supplementary Figure S3.**

Pathway analysis of a) PaCa3, b) PaCa44, c) MiaPaCa2, and d) PC1J CSCs compared to relative P cells. Coloured nodes indicated active/suppressed status. Green and red edges indicated positive and negative Z-score, respectively.

**
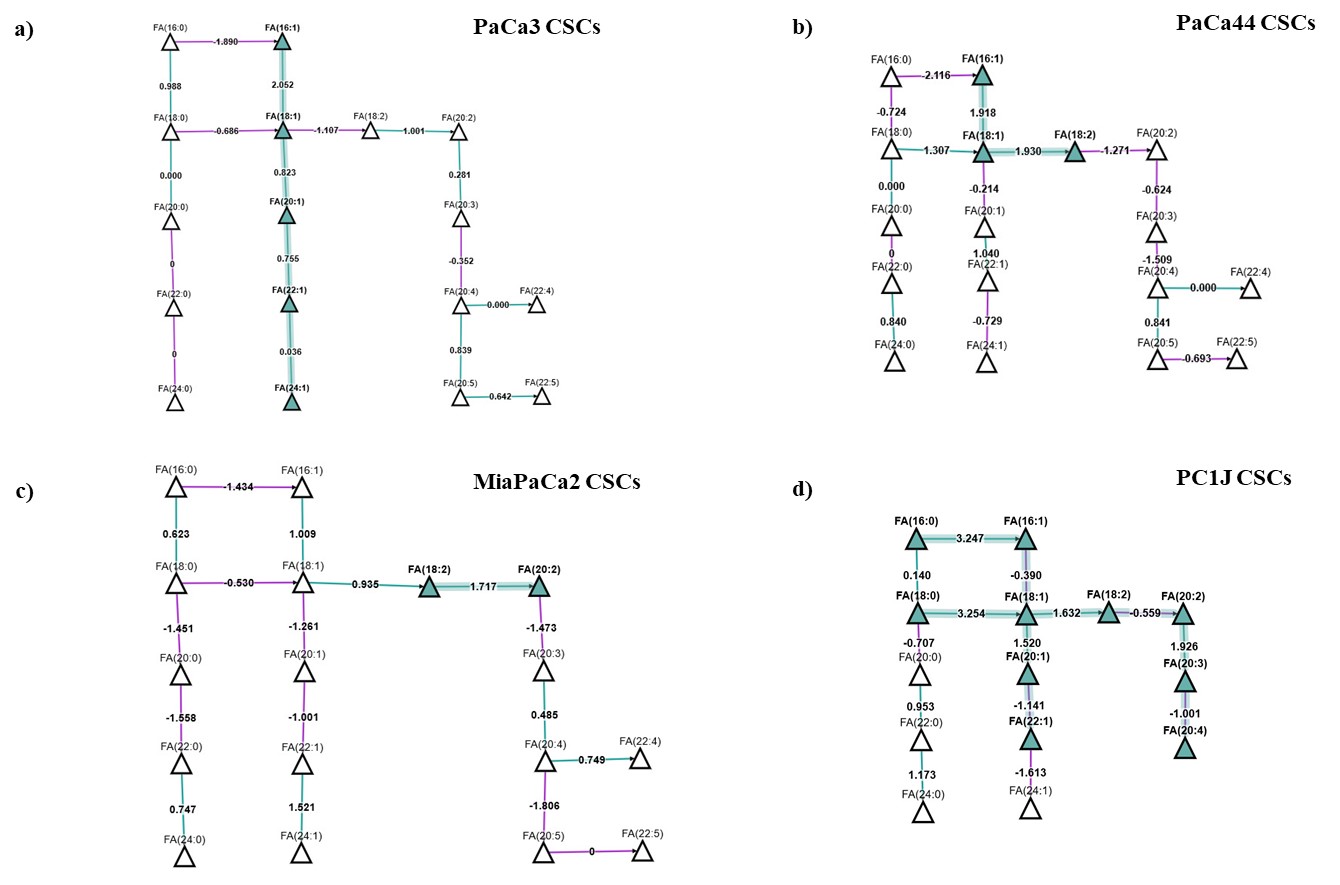
**

**Supplementary Figure S4.**

FA pathway analysis of a) PaCa3, b) PaCa44, c) MiaPaCa2, and d) PC1J CSCs compared to relative P cells. Coloured nodes indicated the most active/suppressed status. Green and purple edges indicated positive and negative Z-score, respectively. The reactions with an active status are highlighted.
