# Supplementary material for: Integrated lipidomics and proteomics reveal cardiolipin alterations, upregulation of HADHA and long chain fatty acids in pancreatic cancer stem cells
Source: Sci Rep. 2021 Jun 24;11:13297. doi: 10.1038/s41598-021-92752-5 (PMC8225828; doi:10.1038/s41598-021-92752-5)
Supplement: Supplementary file 2 — Supplementary Information 2. [file 41598_2021_92752_MOESM2_ESM.docx]

Supplementary Table legends

**Supplementary Table S1.** Dysregulated proteins of PCSCs identified by LC-MS/MS.

**Supplementary Table S2.** Significantly enriched GO terms and pathways of PCSC dysregulated proteins.

**Supplementary Table S3.** Dysregulated lipids of PCSCs identified by LC-MS/MS.

**Supplemental Table S4.** Forward and reverse primers used in Real-Time PCR analysis.

**Supplemental Tab S5.** Antibodies used for Western Blot analysis.
